# An initial but receding altercentric bias in preverbal infants' memory

**DOI:** 10.1098/rspb.2023.0738

**Published:** 2023-06-14

**Authors:** Velisar Manea, Dora Kampis, Charlotte Grosse Wiesmann, Barbu Revencu, Victoria Southgate

**Affiliations:** ^1^ Department of Psychology, University of Copenhagen, København 1353, Denmark; ^2^ Max Planck Institute for Human Cognitive and Brain Sciences, Leipzig 04103, Germany; ^3^ Department of Cognitive Science, Central European University, Vienna, 1100, Austria

**Keywords:** perspective-taking, altercentric bias, infants, social cognition, memory, false-belief task

## Abstract

Young learners would seem to face a daunting challenge in selecting to what they should attend, a problem that may have been exacerbated in human infants by changes in carrying practices during human evolution. A novel theory proposes that human infant cognition has an altercentric bias whereby early in life, infants prioritize encoding events that are the targets of others’ attention. We tested for this bias by asking whether, when the infant and an observing agent have a conflicting perspective on an object's location, the co-witnessed location is better remembered. We found that 8- but not 12-month-olds expected the object to be at the location where the agent had seen it. These findings suggest that in the first year of life, infants may prioritize the encoding of events to which others attend, even though it may sometimes result in memory errors. However, the disappearance of this bias by 12 months suggests that altercentricism is a feature of very early cognition. We propose that it facilitates learning at a unique stage in the life history when motoric immaturity limits infants' interaction with the environment; at this stage, observing others could maximally leverage the information selection process.

## 1. Introduction

While many species learn by observation, human infants are the most prolific such learners [1,2], a feat that is undoubtedly facilitated by teaching [3]. Yet, information is available when social actors are not actively communicating, as their attentional cues carry information about the environment that may be relevant for learning. The emergence of learning devices that ensure the young learner can align their attention with knowledgeable conspecifics [4] may have been especially important given changes in carrying practices during human evolution that likely led to periods of time during which the young infant was physically separated from its mother [5,6]. Recently, it has been proposed that very early in life, while infants are still relatively immobile, an altercentric bias was selected for sampling and encoding of information [7].

Although it has long been held that infants are egocentric [8], evidence for egocentrism comes mainly from older infants and young children. For example, 3-year-olds' tendency to predict someone else's actions based on the child's own knowledge rather than the other's knowledge [9] was classically interpreted as an inability to overcome egocentricity [10], which itself depends on the maturation of inhibitory control [11]. The difficulty in overcoming one's own perspective when reasoning about the perspective of others is also documented in adults [12,13], suggesting that egocentric interference persists throughout life.

However, analogous work with preverbal infants suggests that unlike three-year-old children, infants as young as six months can correctly predict the action of another agent who has outdated information. Across numerous studies using nonverbal tasks, infants seemingly ignore their own perspective and form expectations about others' actions based rather on what the other has seen [14–19]. Arguably the biggest challenge that this infant data presents is how to account for the apparent absence of egocentric influence when infants have notoriously poor inhibitory control [20]. Various accounts have attempted to address this challenge in different ways, but common to most is the assumption that it is the nonverbal nature of the task that allows infants to take others’ perspectives, or to appear as if they can [21–25].

Recently, a novel account proposed that it is not the nature of the task, but rather the nature of human infant cognition that may circumvent the need to manage conflicting perspectives [7]. Informed by work suggesting that both adults and infants experience interference from others' perspectives [13,15,26,27], this account proposes that infant cognition has an altercentric bias that prioritizes the encoding of information derived from another's perspective over events witnessed in the absence of other agents [7]. The term altercentric describes how our own perception and resulting cognitive processing can be altered by the presence of others [26]. Several studies have measured behaviour in situations where participants must respond based on their own perspective, but someone with a conflicting perspective is present [13,15,28]. For example, participants are slower to respond to confirmation of their own perspective when the other's perspective differs [29], and faster to detect the presence of a ball in a scene when another agent should believe the ball to be there, even if the participant themselves should not [15,30,31]. These studies suggest interference from a spontaneous encoding of the other's perspective. Altercentric interference is also present in infants, in similar paradigms. For example, if another agent should expect a ball to be hidden behind an occluder, infants seem to share this expectation even when they themselves have seen the ball depart [15]. In another paradigm, how long 14-month-olds search for an object in a box is influenced by the agent's perspective: if that agent should think that there is a ball remaining in the box, infants will search longer than if that agent shares the infant's perspective that no ball remains in the box [32].

The altercentric hypothesis proposes that young infants can track others' perspectives without the need to manage conflicting perspectives because the two perspectives do not exert a competing influence on infants’ memory. For older children and adults, their own and the other's perspective produce a conflicting representation about the location of the same object. However, for infants, the targets of others' attention are hypothesized to be encoded and remembered better than events that occur in the absence of the other, and thus conflict is reduced or avoided [7]. Such a bias may have been selected for because for young infants whose ability to act on the world themselves is limited, attention to input selected by others may be most valuable. Drawing on a large body of work suggesting that in the first year of life, infants do not have a distinct representation of the self [33], it is proposed that a key feature of early development that fosters an altercentric bias is the initial absence of self-awareness. The altercentric hypothesis proposes that the absence of a distinct self-representation is associated with a relatively weaker memory for events that the infant sees alone than events that are cued by others’ attention. When there is a conflict of perspectives, memory for an event that was not co-witnessed with another agent cannot compete with memory for an event that is observed by another agent.

Thus, an altercentric bias arises in young infants as a result of both the tendency for spontaneous encoding of others' attention and the initial absence of self-representation. This prioritization of what is encoded in the other's presence creates not merely an altercentric interference in which the other's perspective is encoded as well as the participant's own, but an altercentric bias in which the other's perspective is encoded instead of the participant's own. Thus, the difference—in terms of altercentric influence between infant and adult cognition—is not simply one of degree. It is proposed that this bias will serve to constrain infants' attention to information selected by others. Built on a gaze-following foundation shared with other species, this bias highlights high-priority *learning* targets for human infants.

The altercentric hypothesis thus makes a straightforward prediction that infants will misremember an object at a location where it was co-witnessed with another agent, rather than at a location where the infant subsequently sees the same object alone. Such a situation is analogous to the classic change-of-location false-belief event in which an agent is absent when a target object is moved to a new location and so has a false belief about the location of the object. However, rather than testing where the infant expects the agent to search, we test where infants remember the object to be. This is analogous to the non-verbal equivalent of the memory control question that 3- and 4-year-olds are asked in False-Belief tasks to ensure they know the true location of the object [9].

## 2. General methodology

**Figure 1.**
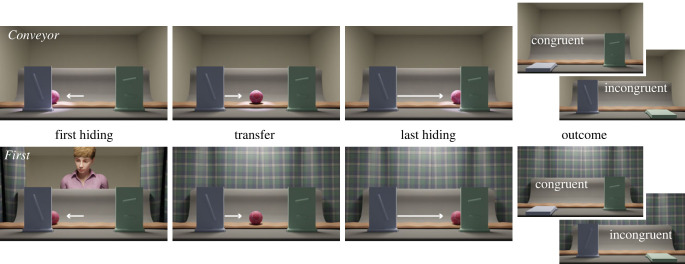
Still frames depicting the important events from the two principal conditions of interest in which the second hiding events are identical. Top: In the non-social *Conveyor* condition the ball in the centre of the scene is first transported behind one occluder (first hiding) and then transferred behind the second occluder (last hiding). In the *Hand* condition, a hand transports the ball. Bottom: In the social *First* condition, an agent witnesses the first hiding event, and then the curtains in front of her close and infants witness the second hiding alone. In the *Both* condition, the curtains only close after the last hiding event. At outcome either the first or the second occluder is lowered, always revealing the absence of the ball.

In each reported condition, 8- and 12-month-old infants saw a three-dimensional animation (see Procedure and Stimuli in the electronic supplementary material for details) where a ball was transported first behind one occluder, and later behind a second occluder (figure 1). The younger age groups played briefly with a real-life identical ball before entering the testing room [34] (see electronic supplementary material for details). Infants’ memory for the location of the object was tested by lowering one of the occluders in each trial and revealing the object to be absent. The last frame of the reveal animation was frozen, and looking time at this static, empty, scene was the dependent measure. The outcome of any given trial was either congruent or incongruent with ‘reality’: on congruent trials, the occluder lowered was the one behind which the object *should* be; on incongruent trials, the occluder lowered was the one behind which the object *should not* be. A difference in looking time between outcomes (incongruent–congruent) is interpreted as a violation of expectation (VoE). Importantly, infants saw the exact same outcome in each trial pair (e.g. in both trials of a pair, the left-hand occluder might be lowered to reveal no ball), but the outcome was congruent with reality when the infant had last seen the object disappear behind the right-hand occluder, but incongruent with reality when infants had last seen the ball disappear behind the left-hand occluder (see Counterbalancing in the electronic supplementary material, text). Consequently, any difference in looking time towards the outcome must be due to infants' memory of the ball's location—and whether or not their expectation of the ball's location is violated. The procedure, original stimuli 3d files, data analysis model, data and stimuli examples are available on OSF at https://osf.io/pqxv5/.

## 3. Measures

We preregistered both total looking time and the duration of the first uninterrupted look [35,36] as dependent measures. *Total looking time* describes looking anywhere inside the screen-space with interruptions no longer than 2 s, or for a maximum of 20 s from the last frame of the reveal animation (see electronic supplementary material for details on the decision). The *first look* measure is the duration from the last frame of the reveal animation until the first instance of the infant looking outside the screen-space for any amount of time. The first look dependent variable is thus a subset of total looking time. The two measures are highly correlated at the level of the entire dataset (*r* = 0.8, *p* < 0.001). We preregistered both measures as they carry potentially different tradeoffs. Total looking time has arguably more stability to random looking-away events, as it has the 2 s buffer. First look may be a more sensitive measure of violation of expectation, as it records only babies' initial stare at the reveal event.

All looking times were log-transformed prior to analysis, as recommended for looking time studies [37].

## 4. Coding

Looking time was coded offline by the first author and double-coded in its entirety by a naïve second coder. Inter-rater reliability was over *r* > 0.95 and the final analysis is based on the coding by the first author.

## 5. Experimental conditions

The first 8 conditions report data from 8-month-olds. We chose 8-month-olds as our initial target age group for testing the altercentric hypothesis because previous data suggest altercentric interference at 7.5 months [15], and it is an age well before the documented onset of self-representation. We preregistered a sequential testing strategy to first obtain evidence for object memory at the ball's last location when no agent is present (*Hand* and *Conveyor* conditions) and then contrast this with the critical experimental condition in which the first hiding event is co-witnessed with an agent (condition *First*, with the False Belief logic). A control condition where the agent witnesses both locations (*Both,* analogous to True Belief) was also run. These conditions were preregistered and the description of the testing protocol, testing materials, and planned analyses can be found here: #33255 | As Predicted: https://aspredicted.org/37uh4.pdf. Several additional exploratory conditions were also included (*Transfer* and *Last*) to further understand findings from condition *Both*. The procedure and data analysis were identical to the previous conditions. We also ran identical replications of the critical condition, *First*, and the mirror-symmetrical *Last*. We ran replications of these two conditions to increase our confidence in our data, and because it is these conditions that we then ran with two groups of 12-month-olds (#71401 | As Predicted: https://aspredicted.org/88ij3.pdf) to test for the onset of the transition away from altercentrism. By 12 months of age, infants are becoming more mobile, and precursors of self-representation may be visible [38].

Based on simulations by Oakes [39], each of the conditions in the 8-month-old group included 32 infants (*Last* replication had 31). The 12-month-old groups are composed of 48 infants, resulting in a total of 351 infants. All participants were recruited from a database of infants whose parents had volunteered them for participation.

## 6. Data analysis

**Figure 2.**
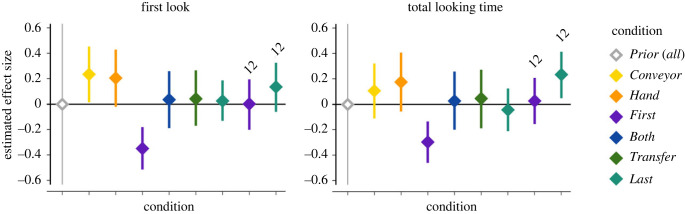
Estimated effect of trial outcome (Incongruent–Congruent) by condition and age group: priors and posteriors. Diamonds represent means, error bars represent the 89% credible interval around the mean. Grey: prior distribution (equal, *a priori*, for all conditions); coloured: posterior distributions by condition. For the 8-month-old group, the direct replications are merged with the original studies (conditions *First* and *Last*).

In the main text, we report the output of the preregistered Bayesian analysis. Bayesian models have several advantages: they do not lose information due to averaging of trials to satisfy the independence assumption; they do not rely on single point estimates, but provide the full (posterior) probability distribution and do not suffer from the problem of multiple comparisons, which one has to correct for when doing frequentist analyses [40]. This is particularly relevant in a complex design such as ours, where we need to estimate not only the effect of outcome within a single condition but also how the effect of outcome varies across multiple conditions and age groups. Finally, it has advantages in clarity, as the entire output of the statistical analysis can be read in one single figure (figure 2). For a discussion of the approach, see Bayesian model in the electronic supplementary material. Commented scripts for reproducing or altering the model can be found on the OSF repository: https://osf.io/pqxv5/. For frequentist statistics, see the electronic supplementary material as well.

In line with the preregistration, we fitted a multilevel Bayesian linear regression model to the log-transformed first-looks and to the log-transformed total looking time for both age groups. The model assumes that the logarithm of looking times produced in any given trial is sampled with noise from a normal distribution whose mean is a linear function of (i) the subject producing that particular measurement; (ii) the outcome–condition combination underlying that measurement; (iii) the trial pair; and (iv) the outcome order in that pair. This allowed us to assess (i) whether there is a difference between congruent and incongruent outcomes in each condition and, if so, in which direction; (ii) the pairwise difference between conditions; and (iii) whether there is an outcome–condition interaction at the level of the entire dataset. The two identical replications of the *First* and *Last* conditions in the 8-month-old age group were merged with their corresponding original samples. The analyses were performed in R 4.1.2 (R Core Team, 2021), using the rethinking package 2.01 [40].

For all questions of interest, we report 89% *credible* intervals (CI) [40,41]. Unlike confidence intervals, these intervals have a straightforward interpretation: with 89% probability, the true value lies in this interval—provided our modelling assumptions are justified. Thus, when we are interested in ruling out a null value, and the 89% credible interval excludes this value, we may conclude that the null value is excluded with 94.5% probability (5.5% for each tail). While we rely on this threshold for testing our hypotheses against the data, it is important to keep in mind that uncertainty is a continuous measure and should be discretized for binary decisions with caution.

In the cases where we ran identical replications (*First*, *Last*), we report the identical conditions as single conditions since each individual tested provides us with additional evidence. In the frequentist analyses reported in the electronic supplementary material, the replications are separated.

## 7. Location memory with and without agency

On the basis of previous work, we expected infants of this age to remember the last location of the object when no other perspective is involved. In most studies in which location memory in young infants has been demonstrated, a hand manipulates and places the object [36,42–44]. Hands belong to agents, thus giving away cues of agency. While a hand by itself does not give away spatial cues of perspective, infants may link hands to agents and agents to perspectives. As the critical condition (called *First*) was intended as a strict contrast between a first co-witnessed displacement and a second ‘witnessed-alone’ displacement, the ideal second displacement would be without perspective—or agency—cues. Thus, two initial conditions depicted either a hand (agency) or a conveyor belt (no agency) transporting the object behind each occluder. Should infants evidence similar sensitivity to the incongruent outcome in both conditions, in the critical co-witnessing condition (*First*), the object will be transported using the conveyor belt to minimize agency cues for the second displacement.

### (a) Material and methods

#### (i) Participants

We tested 32 full-term 8-month-old infants, randomly assigned to each condition (*Hand* mean age: 235 days, range: 215–159 days, s.d. 11, 24 girls; *Conveyor* mean age: 236 days, range: 208–252 days, s.d. 12, 14 girls). Over both conditions of this initial study, a further 50 infants were excluded because of fussiness (*n* = 14), inattentiveness during the object transfer (*n* = 24), experimenter error (*n* = 9), reaching the 20 s looking time during the measurement period for both trial of a trial pair (*n* = 2) and parental interference (*n* = 1). For the *Hand* condition, all 32 infants contributed both pairs; in *Conveyor*, 30 infants contributed both pairs and 2 infants contributed 1 pair (in both cases the first pair)^1^.

#### (ii) Stimuli

Infants observed movies^2^ in which either a hand or conveyor belt transported a ball behind occluders (figure 1, top). In 4 familiarization trials, the hand first grabbed the ball from the centre of the scene and placed it behind one of two occluders (*Hand* condition), or the ball was transported by the conveyor belt behind one of two occluders (*Conveyor* condition). At the end of each of the familiarization trials, infants saw one of the occluders lowered. The aim of the familiarization was to expose infants to reality congruent outcomes that included both the ball present and absent.

Next, on four test trials, the hand or conveyor belt moved the ball behind one of the occluders and then transferred it behind the other. Each test trial ended with one of the occluders lowered, each time revealing no ball. Thus, the outcome was either congruent (videos, *Conveyor*: https://osf.io/764sf*, Hand*: https://osf.io/kqtj5) or incongruent (*Conveyor*: https://osf.io/qgacn; *Hand*: https://osf.io/xak3h) with reality. To best match the additional movements that the hand introduces, the conveyor belt trials included a spotlight that moved around the scene, matching the same parts of the screen occupied by the hand's action. The spotlight's movement was asynchronous to the conveyor belt, so as not to give the impression that it *caused* the conveyor belt to move.

### (b) Results

For descriptives, see electronic supplementary material, table S1 and figure S1.

#### (i) Within-participant outcome differences

Participants’ first looking durations were overall higher for the Incongruent compared to the Congruent outcome. In the *Conveyor* condition, the mean effect size = 0.220, 89% [40] credible interval: [0.001, 0.442]. In the *Hand* condition, the effect size is smaller, and the credible interval (hence CI) does not exclude 0 (mean = 0.191, 89% CI: [–0.034, 0.412]). Total looking time posteriors show a similar pattern to those estimated for first looking durations although neither credible interval excluded 0 (*Conveyor*: mean = 0.098, 89% CI: [–0.136, 0.330]; *Hand*: effect size mean = 0.166, 89% CI: [–0.058, 0.399]).

#### (ii) Outcome–condition interaction

The Incongruent–Congruent manipulation did not affect the two conditions differently: for first looks, the mean of the estimated interaction effect was = 0.028, 89% CI: [–0.283, 0.349]; for total looking time, the effect mean = −0.068, 89%CI: [–0.392, 0.254].

### (c) Discussion

These results confirm that, with first look as the dependent measure, 8-month-olds looked longer to the incongruent than congruent outcome, suggesting that they remembered the last location of the ball. Furthermore, location memory was not modulated by whether infants saw the hand or conveyor belt transporting the object. Thus, in all subsequent conditions, infants observed the conveyor belt transporting the ball.

For total looking time, we noticed that, across the two conditions, close to a third of participants had at least one trial out of the 4 where they looked at the screen for close to 20 s in total (for example, *n* = 18 looked 18–20 s). This may have introduced an artificial ceiling to the total looking time measure, whereas first looks are, by definition, much less affected. Since the cutout was preregistered and the ceiling is only going to affect results where the effect is relatively small, we kept it for the rest of the conditions with the same age group.

## 8. Perspective cue on first versus both witnessed locations

Having confirmed that 8-month-olds are able to remember the last location in which they have seen the ball, we moved on to probe the main claim of altercentric hypothesis: that the presence of an agent during the first hiding event will fundamentally change infants' memory for the ball's location. The ‘*First’* condition provides the critical test because it predicts the opposite pattern of looking time from that of the non-social object displacement reported above. The preregistered prediction was that infants will misremember the ball at the first location, and thus look for longer at the outcome with the ball's absence at the first location (congruent with reality) than at the second (incongruent with reality). In other words, if infants misremember the object at the hiding location co-witnessed with the agent, the congruent outcome should be more surprising for them than the incongruent outcome. In a further ‘*Both’* condition, another group of infants observed the same ball displacements, but the agent observed both the first and second displacement (both hiding locations were co-witnessed). The events are equivalent to those of a ‘True Belief’ control condition of a Theory of Mind task, but without the agent returning at the end. Since the second location is where both the infant and the agent last saw the object, we expected that infants would look for longer at the absence of the object at the second location, as in the non-social conditions (*Hand* or *Conveyor*).

### (a) Material and methods

#### (i) Participants

As before, we had 32 participants per condition (*First*, *Both*, *First replication*). In the *First* condition, the average age of participants was 245 days (232–266, s.d. 9, 10 girls). In the *Both* condition, the mean age was 248 days (240–259, s.d. 11, 14 girls). In condition *Both*, the mean age was 248 days (240–259, s.d. 11, 14 girls). For the crucial *First* condition, we also ran an identical replication. The average age of the replication was 247 days (242–256, s.d. 6, 12 girls).

A further 7 infants were excluded in *First*, for fussiness (*n* = 2), inattentiveness (*n =* 1) experimenter error (*n* = 3), and reaching the looking time cap for both test trials of the first pair (*n* = 1). In *Both*, 3 infants were excluded due to inattentiveness. In *First* *replication* 6 infants were excluded for inattentiveness (*n =* 5) and reaching the cap (*n* = 1).

Of the 32 infants in *Both*, 27 contributed both trial pairs (see electronic supplementary material for exclusion criteria adjustments). For the *First* condition, 29 infants contributed both pairs and 3 infants contributed only the first pair; in *Both*, 27 contributed both trial pairs and for the *First Replication* condition, 26 infants contributed both trial pairs.

#### (ii) Stimuli

In the familiarization trials an agent was present in the background and visually tracked the ball as it was transported by the conveyor belt. On test trials, infants saw the same sequence of ball movements as in *Conveyor*, but these new conditions differed in how much of the ball's movements the co-witnessing agent observed. In *First*, the agent appeared prior to the first displacement and observed the ball as it was transported behind the first occluder. The curtains then closed to hide the agent, after which the ball emerged from behind the first occluder and was transported behind the second occluder (video, Congruent outcome example: https://osf.io/zkgyu or figure 1, bottom). The second displacement was thus witnessed by the infant alone. In *Both* (video), the agent was revealed prior to the first displacement and observed the ball as it was transported by the conveyor belt behind the first occluder and then the second, before the curtains closed to hide the agent. Both displacements were thus witnessed by the infant and the agent. As before, one of the occluders was then lowered to reveal the absence of the ball at either the first or second location. The last frame of the video was paused until the infant looked away for 2 consecutive seconds or until 20 s had elapsed.

### (b) Results

#### (i) Within-participant differences

As predicted, the direction of the effect was reversed for the *First* condition (and its identical replication), with looking time to the Incongruent outcome shorter than that to the Congruent outcome. With first looks, the mean estimated effect size was −0.330, 89% CI: [−0.498, −0.162]; total looking time mean = −0.282, 89% CI: [−0.454, −0.118]. The negative values indicate the direction, as for all conditions we look at Incongruent – Congruent. Here, looking time to Congruent was longer. In the *Both* condition, the mean estimated effect size included 0 with both measures: first looks mean = 0.032, 89% CI: [−0.207, 0.271]; total looking time mean = 0.019, 89% CI: [−0.214, 0.249]. See electronic supplementary material, table S1, for descriptives.

#### (ii) Outcome–condition interaction

Condition *First* is different from all previous conditions with both first looks and total looking time. For *First* versus *Conveyor*, the mean of estimated interaction effect, with first looks = 0.550, 89% CI: [0.277, 0.823], and for total looking time, the mean = 0.380, 89% CI: [0.094, 0.663]. In *First* versus *Hand* first looks = 0.521, 89% CI: [0.240, 0.801] and total looking time = 0.448, 89% CI: [0.165, 0.730]. *First* versus *Both* were also different, with a mean = 0.362, 89% CI: [0.069, 0.656] for first looks and a mean = 0.301, 89% CI: [0.015, 0.582] for total looking time.

### (c) Discussion

The results of the *First* condition are consistent with our preregistered prediction that co-witnessing the first hiding with another agent would reverse infants' expectation about the location of the ball (figure 2 & electronic supplementary material, figure S1). That infants looked longer at the absence of the ball in the first hiding location rather than in the second thus reveals the predicted memory error when the perspectives of the infant and the agent diverge; and it suggests that infants may prioritize encoding the scene as it was when it was co-witnessed with the on-screen agent (see electronic supplementary material for a separate reporting of the replication).

Nonetheless, the finding from the *Both* condition did not conform to our prediction that infants would look longer to the Incongruent outcome, as they did in the non-social conditions. This was predicted because this outcome is both incongruent with what the infant has last seen, and with what the co-witnessing agent has last seen.

A possible explanation for why infants did not have a stronger expectation of the object at the final location than at the first location in the *Both* condition could be that co-witnessing led the infants to encode the object at both locations. The possibility of memory traces in multiple locations has previously been proposed as an explanations for infants' apparent memory failures on classic tasks of object permanence [45,46]. If so, we reasoned that a situation in which the agent and the infant only co-witnessed the final location should generate in infants a clearer expectation of the object at its last location.

## 9. Perspective cue on the last location only

We ran two exploratory conditions in which we varied the timing of the agent's appearance in the scene, and thus what she co-witnessed. In one, the agent appears only for the second part of the object's transition from first to second hiding locations (*Last*), which aims to ensure that the infant and agent have only co-witnessed the object at its last location. However, the sudden appearance of the agent in the middle of the object's transition from first to second location could potentially disrupt infants' tracking of the ball. We therefore also ran a version in which the agent appears once the object disappears behind the first occluder so that the infant and the agent do not co-witness the first hiding, but they co-witness the entirety of the ball's transition from first to second location (*Transfer*).

### (a) Materials and methods

#### (i) Participants

The average age for the 32 participants in the *Transfer* condition was 245 days (230–256, *s.d.* = 9; 15 girls). We ran—and later identically replicated—the *Last* condition, with *n* = 32 in the original and *n* = 31 in the replication. For the first run the mean age was 251 days (244–254, *s.d.* = 4; 13 girls) and for the replication the mean age was 247 days (236–260, *s.d.* = 6; 18 girls). A further 9 infants were excluded in *Transfer* because of inattentiveness during the object transfer (*n* = 7), fussiness (*n* = 1) and reaching the 20 s cap in both measurement periods of the first pair of trials (*n* = 1). For the *Last* condition and its replication, 32 infants were excluded. The higher number of exclusions was due to a counterbalancing error in the outcome's side that was discovered after running the participants (for details, see the *Counterbalancing error in Last* section in the electronic supplementary material). In addition, 12 infants were excluded due to inattentiveness (*n* = 12) and one infant for reaching the maximum looking time cap in both trials of the first pair (*n* = 1). In the *Transfer* condition, 28 infants contributed both pairs and 4 infants contributed only the first pair. For the *Last* condition and its replication, 52 infants contributed both pairs, and 11 infants contributed only the first pair.

#### (ii) Stimuli

Familiarization events were identical to those before. In the *Transfer* condition (see the online video, Incongruent outcome example: https://osf.io/dcm6f), infants observed test trials in which the agent appears after the ball has disappeared behind the first occluder but before it emerges to begin its transition to the second location. While the agent appears when the ball is hidden in its first location, she only attends to the ball—and tracks its movement—from the midpoint in its transition to the second hiding location. In the *Last* condition (video: https://osf.io/t8mx3), infants observed test trials in which the agent appears as the ball pauses briefly during its transition from the first to the second hiding location. In both conditions, the curtains close to hide the agent before one of the occluders is lowered to reveal the absence of the ball at either the first or second location.

### (b) Results

#### (i) Within-participant differences

In both of the exploratory conditions the posterior on the effect size was centred close to zero with both measures. In condition *Transfer*, the mean estimated effect size for first looks was 0.040, 89% CI: [−0.200, 0.262] and for total looking time 0.042, 89% CI: [−0.180, 0.264]. Condition *Last* (identical replication included) had the mean = 0.023, 89% CI: [−0.142., 0.190] and total looking time mean was −0.040, 89% CI: [−0.206, 0.127].

#### (ii) Outcome–condition interaction

The two new conditions do not differ from each other (mean = −0.083, 89% CI: [−0.373, 0.212]) and neither is different from the condition *Both* (versus *Transfer*, mean = −0.023, 89% CI: [−0.344, 0.302]; versus *Last*, mean = −0.060, 89% CI: [−0.337, 0.218]). They are both different from condition *First* (versus *Transfer*, mean = −0.325, 89% CI: [−0.039, −0.609]; versus *Last*, mean = −0.241, 89% CI: [−0.002, −0.478]) (for total looking time, see OSF).

### (c) Discussion

Thus, these additional conditions did not shed light on the null result in the *Both* condition, instead yielding further evidence that the presence of a co-witnessing agent who, together with the infant, observes the ball at its final location, does not lead infants to have an expectation that the ball should be present at this final location. This is puzzling because *a*) in the absence of an agent (*non-social* conditions), infants evidence an expectation that a ball they see disappear behind a second occluder should be present behind that occluder and *b*) in the presence of an agent who co-witnesses the ball only at the first location (condition *First*), infants generate a clear expectation that the ball should be behind the first occluder.

We considered whether in the crucial condition, *First,* the agent's disappearance after the first hiding may have distracted infants from the ball's second displacement. Thus, we coded and compared how much of the ball's second transfer infants witnessed in this *First* condition (when the agent disappeared before the second transfer) compared to the identical movement in the non-social *Conveyor* condition (when no agent was present for either the first or the second transfer; table 1). This analysis revealed that infants spent most of the 4 second transition period watching the ball in both the non-social (82%, *s.d.* 7.3%) and *First* conditions (81.5%, *s.d.* 8%), indicating that the agent's disappearance did not change infants' visual attention to the subsequent transition from first to second location. Thus, despite infants spending the majority of the second transfer focused on the movement of the ball, they did not remember its final location when its first location was co-witnessed. Furthermore, while in the condition *First*, infants spent less time watching the ball during its first hiding (61.6%, s.d. 20.9%) than its second hiding (81.5%, s.d. 8.0%) because there is also an agent on-screen, it is at the first location that they appear to remember it. This indicates that infants’ memory of the ball in its first location was not due to increased visual attention to its disappearance.

**Table 1.** Percentage of time spent looking at the ball, averaged across all infants, during the two hiding events. In some conditions, the agent in the background competes for the infants’ interest.

condition		*Conveyor*	*First*	*Both*	*Transfer*
first hiding (3 s)	agent present?	no	yes	yes	no
	percentage LT	**95.3%** (s.d. 07.9%)	**61.6%** (s.d. 20.9%)	**63.3%** (s.d. 22.8%)	**92.8%** (s.d. 07.2%)
second hiding (4 s)	agent present?	NO	NO	YES	YES
	percentage LT	**82.0%** (s.d. 07.3%)	**81.5%** (s.d. 08.0%)	**67.0%** (s.d. 18.0%)	**59.5%** (s.d. 22.3%)

This observation led us to speculate that it may be infants' attention to the agent and ball relation, rather than just the ball, that predicts where they remember the ball to be. We reasoned that examining looking during the second transfer could inform our null results in the *Both*, *Transfer* and *Last* conditions where the agent is present during the second transfer. To address this, we categorized infants as those who looked predominantly at the ball (object attention) versus those who distributed their attention between the ball and the agent (distributed attention) during the transfer (see electronic supplementary material for details of how infants were categorized). Merging data across the two conditions in which the agent was present for the *entirety* of the second displacement (*Both* and *Transfer*)^3^, we ran a Bayesian Repeated Measures ANOVA in JASP 0.17.1 [41] as an exploratory analysis. We ran an interaction model with Attention (object versus distributed) as a between-subjects factor and Outcome (Incongruent, Congruent) as a within-subjects factor. The null model contained the factors separately. The interaction model (outcome*Attention) had favourable posterior odds ratios under multiple different ways of categorizing infants (see electronic supplementary material for details; cutouts) and follow-up Bayesian paired samples *t*-tests indicate that infants who distributed their attention looked longer at the Incongruent than the Congruent outcome, as we had originally hypothesized for these conditions. Attending only to the ball, on the other hand, did not yield a looking time advantage for either outcome.

This exploratory analysis provides some insight into the puzzling null results from the three conditions where an agent co-witnessed the final transfer together with the infant. Specifically, it indicates that what infants attend to during this second transfer influences what they remember. We do not know why some infants divided their attention between the agent and ball and some focused predominantly on the ball. This difference between infants could plausibly reflect differences in maturation of attention disengagement [47] or differences in the extent to which the infant prioritizes the other's attention. More infants were categorized as ball-lookers (i.e. predominantly looking towards the moving ball and not towards the agent) when there was an agent present for the second hiding (in conditions *Both* + *Transfer*: 33/64) than when there was an agent present for the first hiding (*First* + *Both*: 22/64). This suggests that as a group, more babies divided their attention between the agent and ball, earlier in the trial.

## 10. Older infants

The *First* condition revealed the presence of an altercentric bias in eight-month-olds when the infant and the agent held conflicting perspectives. As this bias was hypothesized to be a particular feature of very early cognition [7], we ask whether the bias remains at 12 months when a) infants are more mobile and versed in their environment and b) precursors of self-awareness may be present. A second aim was to follow-up on the speculation above that 8-month-olds' failure on the *Both, Last* and *Transfer* conditions may be due to challenges with dividing attention later in the trial. As 12-month-olds’ selective attention abilities are likely to be more robust, we hypothesized that they would show evidence of remembering the object in the final location on the *Last* condition, in which the agent only sees the object at the final location. These conditions (*First*, *Last*) were preregistered (#71401 | As Predicted: https://aspredicted.org/37uh4.pdf) as we wanted to change the maximum looking time in the procedure (see below). Additionally, unlike with the exploratory conditions (*Transfer*, *Last* and the replications of *First* and *Last* with younger infants), we had clear predictions in the theory under test.

### (a) Material and methods

#### (i) Participants

For the pair of conditions with 12-month-olds, 96 infants (48 each) were randomly assigned to either the *First 12* or *Last 12* conditions (mean age: 370 days; *s.d.* = 4; 44 girls). A further 23 infants were excluded because of fussiness or inattentiveness (*n* = 15), experimenter error (*n* = 7: subjects were presented with trials with the counterbalancing error) and equipment malfunction (*n* = 1). Of the 96 infants, 94 contributed both trial pairs. We preregistered samples of *n* = 32 for each condition, in line with the previous studies, but chose to test 16 more in each because the data at *n* = 32 were insensitive. The criterion for data sensitivity that we adopted was based on a Bayesian version of the *t-*tests we used for the analysis of our data (see frequentist analyses in electronic supplementary material), where the Bayes factor is between 3:1 and 0.3:1 for either the alternative hypothesis or the null [48,49]. Thus, we randomly selected 16 new trial orders out of the 32 and used the same 16 trial orders in both conditions.

#### (ii) Stimuli

Infants viewed the stimuli of conditions *First* and *Last*. The only difference from the runs with the 8-month-olds was that we extended the duration of the freeze frame to a maximum of 30 s.

### (b) Results

#### (i) Within-participant differences

With the 12-month-olds, the effect size was centred on 0, 89% CI: [−0.187, 0.186] in the *First 12* condition. The total looking time measure was similar at a mean = 0.033, 89% CI: [−0.151, 0.219]. This suggests that, unlike the 8-month-olds, 12-month-olds did not look longer at the Congruent than the Incongruent outcome. In the *Last 12* condition*,* first look mean = 0.146, 89% CI: [−0.034, 0.327], whereas with total looking time, the entire distribution of posteriors was above zero, with the mean = 0.262, 89% CI: [0.082, −0.445]. This suggests that, unlike the 8-month-olds, 12-month-olds looked longer at the Incongruent than the Congruent outcome. We assume that the reason why total looking time was a sensitive dependent measure for 12- but not 8-month-olds is because we raised the maximum cap from 20 to 30 s for the older groups.

#### (ii) Outcome–condition interactions

Is *First 12* different from *Last 12*? Credible intervals for both measures contain zero, 89% CI: [−0.410, 0.115] for first look and 89% CI: [−0.486, 0.028] for total looking time.

#### (iii) Age differences

We were interested in whether 8- and 12-month-olds differed in their looking times to the outcomes of the *First* and *Last* conditions. With *first look* as the dependent variable, the effect of outcome differed between 8- and 12-month-olds in the *First* condition: mean = 0.333, 89% CI: [0.079, 0.585], but not in the *Last* condition: mean = 0.123, 89% CI: [−0.123, 0.356]. With *total looking time*, there was an age difference for both *First* and *Last* conditions (*First* mean = 0.314, 89% CI: [0.065, 0.568]; *Last* mean = 0.302, 89% CI: [0.060, 0.547].

### (c) Discussion

This last set of results suggests that, unlike 8-month-olds, 12-month-olds do remember the last location of the ball on co-witness *Last* trials. However, we found no evidence for the altercentric bias that we observed in 8-month-olds when perspectives diverged on the *First* condition. In fact, 12-month-olds did not show a differential expectation that the ball should be in either location in the *First* condition. We return to possible explanations for this finding below.

## 11. General discussion

The altercentric bias hypothesis proposes that infants' memory for events that are the targets of others’ attention is privileged. A clear prediction of this hypothesis is that, if there is a conflict between what the self and other have experienced, infant memory will prioritize representations derived from tracking the targets of the other's attention. In an object displacement event like that used in the current study, this prioritization of co-witnessed events will lead infants to misremember the location of the object. We first obtained evidence that, with these stimuli, 8-month-old infants would remember the location of an object at its final location. They looked longer towards an outcome that did not reveal the object at the location behind which it was last seen, than at an identical outcome when the object had not last been seen behind that occluder. Given that the outcomes were identical across trial pairs, and the only factor that varied was whether the object should be behind the occluder, we interpret longer looking towards the absence of the object on Incongruent outcomes as reflecting infants' memory for the location of the ball. Next, we asked whether we could reverse infants’ expectation of the ball's location by including an agent who co-witnesses the hiding at its first location. In a preregistered condition and replication, we indeed found that 8-month-olds had a stronger expectation that the ball should be in the first location than the second, even though in both conditions they attended equally to the second displacement. Coding of infants' attention to the ball (table 1) indicates that 8-month-olds attended to the second displacement and did so to the same degree whether or not (*First* versus *Conveyor*) an agent disappeared prior to this event. This strongly suggests that infants were not simply distracted by the agent's disappearance and failed to notice the ball moving to its final location. Rather, they watched the ball moving to its final location, but expected it to be in the first, co-witnessed location. We interpret this as indicating that if there is a conflict in perspectives, 8-month-old infants remember better what they co-witness with another agent than what they subsequently witness alone, as predicted by the hypothesis [7].

12-month-olds did not show this altercentric bias. However, while the bias is no longer present, 12-month-olds still do not remember the last location of the object if the first location (but not the last) was co-witnessed. One possibility is that 12 months is a point of transition where some infants are now less susceptible to the others' perspective, but some remain so, and thus group data reflect both groups of infants such that it appears that they have no strong expectation as to the ball's location. Originally conceived, the altercentric bias hypothesis was suggested as a learning aid for a life-history stage where, due to motoric immaturity, infants are largely observers and encoding events already selected by others could be beneficial. However, as infants become more mobile, they may become better able to select information for themselves. Or, as infant memory undergoes dramatic changes between 8 and 12 months [50], this could shift some infants towards a greater reliance on their own, first-person experience, and less susceptibility to influence from the other's perspective. Finally, and also in line with the original hypothesis, as self-representation emerges, the altercentric bias is hypothesized to recede. While clear evidence of self-representation is found in mirror self-recognition observable from around 18 months, precursors may be found at the beginning of the second year of life [33].

Nevertheless, in 8-month-olds we found an apparent absence of memory for the object's location when the agent witnessed both object displacements. Across three conditions (*Both*, *Transfer* and *Last*), infants did not evidence a greater expectation that the object should be revealed at its last, actual, location. This was unexpected because when the observing agent witnesses everything, there is no conflict in perspectives, and we had predicted that infants would remember the object's last location—as they did in the non-social *Hand* and *Conveyor* conditions. An exploratory analysis of infants' visual attention to the ball versus agent during the transfer of the ball from the first to the second location indicated that infants who distributed attention between the agent and the ball indeed remembered the ball's last location, as predicted. Infants who attended predominantly to the moving ball, in contrast, tended to misremember the ball at its first location—similarly to infants who had only co-witnessed the first hiding with another agent in the critical *First* condition. Although not predicted, this finding is consistent with the core of the altercentric hypothesis: tracking the agent's attention to the ball seems to be the main drive of infants' expectations about the ball's location. These data are also consistent with previous work showing that it is infants' attention to the agent, not the object, that appears to determine what they remember about that object [51].

The data from these conditions are also consistent with a recent review of infant non-verbal Theory of Mind studies, which suggested that while there was evidence for infants’ ability to understand False Belief (similar to our *First* condition), there was little evidence that infants generate correct expectations from True Belief events (similar to our *Both* condition) [52]. Why this should be so is unclear. However, in our study, although we observed variance in attention to the agent and ball on conditions where an agent was present during the first object displacement (*First* and *Both*), more infants attended solely to the object on the second hiding than the first (that is, in *Both* and *Transfer*). It is possible that at 8 months, dividing attention between the agent and ball becomes more effortful as the trial goes on, such that the movement of the ball becomes more difficult to disengage from. That 12-month-olds did remember the last location of the ball under these conditions could be viewed as consistent with this interpretation.

While we used looking time to index object location memory, our data cannot tell us what infants expected to see at the location revealing the object's absence. Historically, different scholars have hypothesized that in similar tasks of object permanence, infants may have memory traces at both locations, which could both contribute to their expectations of object existence [45,46]. Research on memory for object identity suggests that infants younger than 12 months are less sensitive to a change in object identity than to a change in object location [34,53,54], which may be related to knowledge of object action affordance^4^ [55]. Recent research shows that, when tracking a moving object, even adults have only a coarse approximation of the object's form [56]. Thus, it is plausible that what infants represent at the co-witnessed location—or what they generate from tracking the other's attention—is a representation of something relevant at this location, but not necessarily a detailed representation of the object (e.g. *a pink ball*). The fact that it was the group that distributed their attention between agent and ball that seemed to have the stronger expectation of the ball in its actual location is consistent with a representation of ‘something’ rather than a specific object. It is also an open question what infants do or do not encode about the event they see alone. In the *First* condition where infants co-witnessed the first but not the second hiding with the agent, our data show the biggest relative reduction in looking time for the non-co-witnessed location. Insofar as looking time tracks expectation, it is here that infants seem to have the least uncertainty: they act as if they predicted that no ball should be at the last location.

Throughout this paper, we have described the co-witnessing advantage for object memory as a representation derived from infants' attention to the location of others’ attention. Under this view, it is thus not necessary for infants to represent someone's visual attention or perspective as their perspective, in order for infants to benefit from tracking and being cued by this perspective. It is hypothesized that an altercentric bias would serve to constrain infants' attention to events that their adult caregivers have deemed already worthy of attention, and in this way such a bias is proposed to serve an important learning function. Therefore, the hypothesis presents a way for infants to benefit from tracking others’ perspectives without needing to represent their perspective *as such*.

Originally conceived, the altercentric bias hypothesis aimed to explain how young infants could apparently accurately predict where another agent holding a False Belief about an object's location would search, even when the other's representation of the object's location should conflict with the infant's own—a difficult challenge even for much older children. These data offer an answer to this puzzle [57]. Specifically, they suggest that infants can accurately predict where an agent with a False Belief will search because infants have a stronger representation of the object at the location where the other has seen it than they have at the location where they themselves have last seen the object. For young infants, this becomes the first-person representation that also drives how they expect others to behave. If correct, this implies that infants are not thinking about where the other thinks the ball to be, but they are using their—albeit erroneous—representation of the object's location to predict where someone else will likely search. These data reveal something unique about very early human cognition: that far from being egocentric, infants may filter the world through the eyes of more knowledgeable others.

## Data Availability

All raw data, stimuli (including their sources) plus the Bayesian analysis model and the resources needed to be run are accessible at this address on OSF: https://osf.io/pqxv5/. The data are provided in electronic supplementary material [58].

## Endnotes

1 See electronic supplementary material for description of exclusion criteria and a discussion about why after this first pair of conditions we relaxed our needlessly strict criteria.

2 See electronic supplementary material for the details of the presentation procedure.

3 In condition *Last* the agent is revealed during the transfer period, after the ball reached the middle of the screen; the transfer is also one second longer to accommodate the reveal, and has the sound of the curtains played to get infants’ attention to the agent reveal. Given all those differences we did not code this condition as any interpretation would have been difficult.

4 We remind the reader that in the current series of studies infants played briefly with the ball they see in the animations seconds before watching those animations.
